# A Prediction Model of Stable Warfarin Doses in Patients After Mechanical Heart Valve Replacement Based on a Machine Learning Algorithm

**DOI:** 10.31083/RCM33425

**Published:** 2025-06-26

**Authors:** Bowen Guo, Cong Chen, Junhang Jia, Jubing Zheng, Yue Song, Taoshuai Liu, Kui Zhang, Yang Li, Ran Dong

**Affiliations:** ^1^Center of Cardiac Surgery, Beijing Anzhen Hospital, Capital Medical University, 100000 Beijing, China

**Keywords:** warfarin, machine learning, heart valve prosthesis implantation, prediction model

## Abstract

**Background:**

The narrow therapeutic range of warfarin, alongside the response of numerous influencing factors and significant inter-individual variability, presents major challenges for personalized medication. This study aimed to combine clinical and genetic characteristics with machine learning (ML) algorithms to develop and validate a model for predicting stable warfarin doses in patients from Northern China after mechanical heart valve replacement surgery.

**Methods:**

This study included patients who underwent mechanical heart valve replacement surgery at the Beijing Anzhen Hospital between January 2021 and January 2024 and achieved a stable warfarin maintenance dose. Comprehensive clinical and genetic data were collected, and patients were divided into training and validation cohorts at an 8:2 ratio through random division. The variables were selected using analysis of covariance (ANCOVA). Algorithms for predicting the stable warfarin dose were constructed using a traditional linear model, general linear model (GLM), and 10 ML algorithms. The performance of these algorithms was evaluated and compared using R-squared (R^2^), mean absolute error (MAE), and ideal prediction percentage to identify the optimal algorithm for predicting the stable warfarin dose and verify its clinical significance.

**Results:**

A total of 413 patients were included in this study for model training and validation, and 13 important features were selected for model development. The support vector machine radial basis function (SVM Radial) algorithm showed the best performance of all models, with the highest R^2^ value of 0.98 and the lowest MAE of 0.14 mg/day (95% confidence interval (CI): 0.11–0.17). This model successfully predicted the ideal warfarin dose in 93.83% of patients, with the highest ideal prediction percentage found in the medium-dose group (95.92%). In addition, the model demonstrated high predictive accuracy in both the low-dose and high-dose groups, with ideal prediction percentages of 85.71% and 92.00%, respectively.

**Conclusions:**

Compared to previous methods, SVM Radial demonstrates significantly higher accuracy for predicting the warfarin maintenance dose following heart valve replacement surgery, suggesting it has potential for widespread application. However, this study was based on a relatively small sample size and conducted at a single center. Future research should involve larger sample sizes and multicenter data to validate the predictive accuracy of the SVM Radial model further.

## 1. Introduction

Warfarin, the most commonly used vitamin K antagonist (VKA), is the preferred anticoagulant for patients undergoing mechanical heart valve replacement surgery. The 2020 updated American College of Cardiology (ACC)/American Heart Association (AHA) guidelines explicitly recommend VKA for the antithrombotic therapy in patients with mechanical prosthetic valves [1]. However, warfarin has several limitations, including a narrow therapeutic window, considerable dose variability between individuals, and the existence of numerous factors that can influence the anticoagulant effect. Incorrect dosing can increase the risk of bleeding and thromboembolic events, such as gastrointestinal bleeding, cerebral hemorrhage, deep vein thrombosis, pulmonary embolism, and stroke [2, 3, 4]. Therefore, accurate dosing of warfarin is crucial for the efficacy and safety of anticoagulant therapy, with individualized dosing needed to achieve optimal clinical outcomes [5].

Currently, anticoagulation therapy with warfarin usually follows an initial regimen of a fixed standard dose, followed by empirical dose adjustments by the clinician based on international normalized ratio (INR) values. However, before a stable maintenance dose is found, the incidence of anticoagulation-related adverse events such as bleeding and thromboembolism remains high, posing a threat to patient safety [6]. Additionally, patients require regular blood tests to monitor INR levels, which can be inconvenient and require high patient compliance [7].

Several researchers have used pharmacogenomics-based warfarin dosing models to develop new individualized dosing approaches. Most of these use multiple linear regression (MLR) equations with the warfarin therapeutic dose as the dependent variable, and genetic and non-genetic factors as independent variables. Notable examples include the International Warfarin Pharmacogenetics Consortium (IWPC) and Gage models [8, 9]. Although MLR models have been widely used for warfarin dose prediction, their predictive performance varies widely [8, 9, 10]. Validation results across different populations are often unsatisfactory, and their accuracy tends to be lower in specific subgroups. MLR does not take into account the inter-individual variability of warfarin and is unable to effectively utilize patient INR monitoring values for dose adjustments. Advances in research have established that a complex nonlinear relationship exists between the factors influencing warfarin and its dosage [11]. This renders MLR models unsuitable for accurately predicting the stable maintenance dose of warfarin, and more suitable models are required to optimize individual therapies. With the rapid progress in artificial intelligence, machine learning (ML) has sparked widespread research interest in clinical pharmacotherapy and is playing an increasingly important role in personalized medicine, particularly in the selection of drug dosage [12]. ML enables systems to analyze vast amounts of data collected from electronic medical records (EMR) and use advanced statistical and probabilistic techniques to automatically learn from this data. The construction of intelligent and effective predictive models should result in more accurate predictions [13]. Currently, several groups have developed new warfarin dosing models based on ML algorithms. However, most of these studies did not incorporate genetic characteristics, and the predictive accuracy of such models still has to be improved and validated [14, 15, 16, 17, 18]. In 2007 and 2010, the U.S. Food and Drug Administration (FDA) revised the instructions for warfarin and recommended validation of the cytochrome P450 family 2 subfamily C member 9 (*CYP2C9*) and vitamin K epoxide reductase complex subunit 1 (*VKORC1*) genotypes to more accurately guide the individualization of treatment [19, 20]. These changes highlight the importance of genetic information for individualized anticoagulant therapy with warfarin.

In order to overcome the limitations of traditional methods and further improve the prediction accuracy of the model, we developed and validated a prediction model for individualized warfarin maintenance dosage in patients after cardiac mechanical valve replacement based on a large number of clinical and genetic features and combined with 10 ML algorithms.

## 2. Materials and Methods

### 2.1 Subjects

This retrospective study included patients who underwent mechanical heart valve replacement surgery at Beijing Anzhen Hospital, Capital Medical University, and subsequently achieved a stable warfarin dose. Comprehensive clinical data and genetic information was collected from patients who underwent surgery between January 2021 and January 2024.

The inclusion criteria were: (1) northern Chinese population; (2) patients aged ≥18 years on postoperative warfarin anticoagulation therapy, with INR monitoring; (3) achieved warfarin anticoagulation stability, as defined by warfarin usage for ≥3 months, with a consistent dose over the last three consecutive follow-ups (interval ≥7 days), and INR maintained between 1.5–2.5. A dose is considered to be a stable maintenance dose once anticoagulation stability is achieved.

The exclusion criteria were: (1) the patient had severe liver and kidney dysfunction; (2) abnormal preoperative INR values (<0.8 or >1.2); (3) inability to determine genotype; (4) concomitant administration of nonsteroidal anti-inflammatory drugs (NSAIDs); (5) hematologic disorders (e.g., hemophilia, thrombocytopenia, and platelet function disorders); (6) undergoing other concurrent cardiac surgeries (except for the Cox-Maze procedure); (7) patient experienced thromboembolic events, bleeding, or other anticoagulation-related complications, or died during hospitalization or follow-up. Patients who met any of the above criteria were excluded from the study.

The study protocol received ethical approval from the Institutional Review Board of Beijing Anzhen Hospital (Ethics Approval Number: 2020067X), and was conducted in accordance with the Declaration of Helsinki. All participants voluntarily provided written informed consent prior to enrollment.

### 2.2 Sample Size Calculation

To ensure the reliability of the study results, the effect size was set to medium (Cohen’s d = 0.5), the significance level to 0.05 (α = 0.05), and the power to 0.8 (1 – β = 0.08). Based on these parameters, the sample size was calculated using G*Power software (version 3.1; Heinrich-Heine-Universität Düsseldorf, Düsseldorf, North Rhine-Westphalia, Germany). The study required at least 332 valid samples to achieve sufficient power in regression analysis tasks. A total of 413 subjects were actually included in the study, ensuring an adequate sample size.

### 2.3 Clinical Data

Patient clinical data was collected through face-to-face interviews, regular phone calls, and review of the medical records from our hospital information system. These data included demographic characteristics, comorbidities, concomitant medications, preoperative echocardiographic parameters, cardiac function classification (New York Heart Association (NYHA) classification), surgical approaches, stable maintenance dose of warfarin therapy, and steady-state INR. The demographic characteristics included gender, age, height, weight, body surface area (BSA), body mass index (BMI), smoking history, and alcohol consumption history. Comorbidities were defined as chronic conditions or diseases diagnosed before the patient underwent heart valve replacement surgery. These included hypertension, diabetes, cerebrovascular disease, coronary artery disease and atrial fibrillation (AF). The presence of each comorbidity was confirmed by assessing the patient’s medical history and relevant diagnostic test results. Concomitant medications were defined as any medication being taken by the patient alongside the prescribed warfarin regimen, before or after surgery. This included digoxin, amiodarone, statin, and angiotensin converting enzyme inhibitors (ACEI) that could potentially interact with warfarin. A detailed list of these medications was obtained from the patient’s medical records. Preoperative echocardiographic parameters included ejection fraction (EF), left ventricular end-diastolic diameter (LVEDD), left ventricular end-systolic diameter (LVESD), and left atrial diameter (LAD).

Blood samples were drawn from each patient on the morning of the day before surgery and under fasting conditions. The levels of B-type natriuretic peptide (BNP), alanine transaminase (ALT), aspartate aminotransferase (AST), creatinine, triglycerides (TG), total cholesterol (TC), high density lipoprotein (HDL), low density lipoprotein (LDL), INR, fibrinogen, prothrombin time (PT) and other biochemical parameters were determined in the clinical laboratory of Beijing Anzhen Hospital, Capital Medical University, Beijing, China.

### 2.4 DNA Extraction and Single Nucleotide Polymorphism (SNP) Beadchip Assay

A total of 5 mL of venous blood was collected from eligible patients the day before surgery using an EDTA vacuum tube (BD Vacutainer, Franklin Lakes, NJ, USA) and stored at 4 °C before DNA extraction. Genomic DNA was extracted using the Blood DNA System DNA isolation kit (Catalog number: CW2320, CWBIO, Beijing, China) according to the manufacturer’s instructions and stored at 4 °C for subsequent use. DNA quality and purity were assessed by agarose gel electrophoresis and by measuring optical absorbance at A260/A280.

Genotyping of 6 SNPs was performed using the Illumina SNP GoldenGate Assay (Catalog number: WG-2500A-1001, Illumina, San Diego, CA, USA) according to the manufacturer’s instructions. These SNPs were *CYP2C9* (rs1957910), *VKORC1* (rs9923231), *CYP4F2 *(rs2108622), *APOE* (rs7412), *CYP1A2* (rs2069514) and *CYP3A4* (rs28371759). Briefly, 250 ng of genomic DNA was amplified at 37 °C for 20 h, followed by fragmentation and precipitation. The dried pellet was then resuspended and hybridized to the beadchips. These were subsequently incubated at 48 °C for 20 h, washed, and a single-base extension step performed. The beadchips were then stained, washed, coated, and dried. Finally, signal-intensity data were generated by an Illumina BeadArray Reader (Catalog number: BeadArray-1000, Illumina, San Diego, CA, USA). Twenty percent of the samples were selected at random for duplicate genotyping, with 99.8% concordance observed. Inconsistent data were excluded from the final analysis.

### 2.5 Data Preprocessing

The collected data were entered into a Microsoft Excel spreadsheet and saved as a .csv file. During data preprocessing, missing values were first analyzed and the percentage of missing data determined for each variable to ensure completeness. If the missing data for a variable was <5%, the average of multiple estimates was used to fill in. If the missing data for a variable was ≥10%, the variable was removed or filled in using the mean value, depending on the distribution of data. If an individual patient record had >30% missing values, the data for that patient was deleted. Outliers were then detected using box plots and the 3σ rule (i.e., values greater than ±3 times the standard deviation of the mean were considered outliers). If the outliers were input errors, they were manually corrected or excluded. If they were extreme values, the data were excluded as appropriate.

### 2.6 Feature Selection

All clinical and genetic data were included as candidate variables. Analysis of covariance (ANCOVA) was used to evaluate the effect of clinical and genetic variables on the target variable while controlling for potential confounders, selecting variables with statistical significance based on *p*-values (*p* < 0.05) and partial η^2^ effect sizes (η^2^ ≥0.002). Initially, candidate variables were screened by their *p*-values (*p* < 0.05), ensuring that only those significantly related to the target variable were retained. Among the remaining variables, partial η^2^ was then calculated to assess the effect size. Variables with a very small contribution (i.e., η^2^ <0.002) were excluded, as they were deemed to have minimal impact on model performance. Multiple iterations of this process were performed, with each iteration involving the recalculation of partial η^2^ values and *p*-values. In each round, the variables contributing least to model performance were excluded, and the remaining filtered variables were used as input for the ML algorithm, with the stabilized warfarin dose as the output variable. By combining these statistical tools with effect size measures, more scientifically meaningful features were selected to improve model performance and interpretability.

### 2.7 Model Construction

**Fig. 1.**
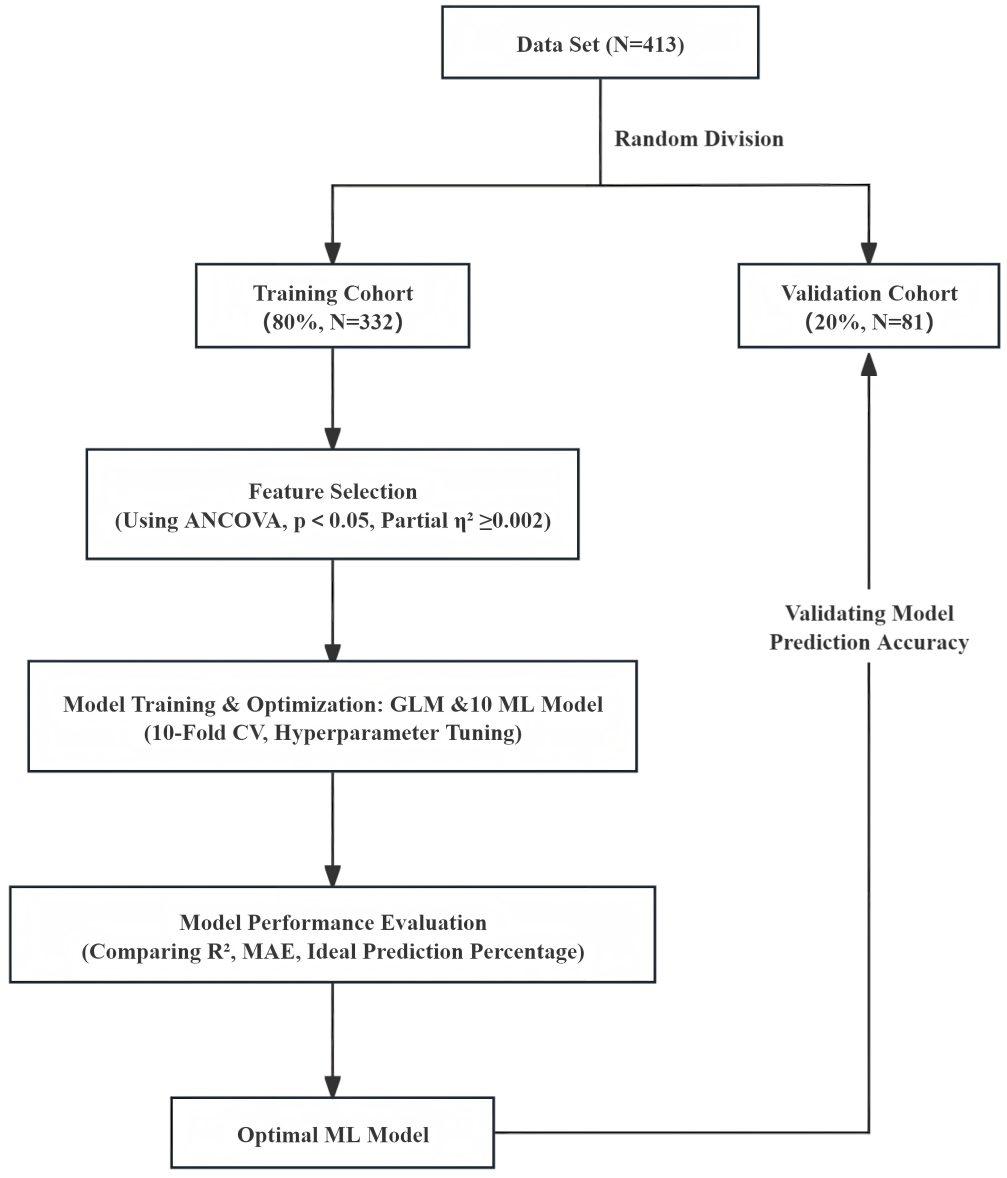
**Flow chart used for modeling**. ANCOVA, analysis of covariance; GLM, general linear model; MAE, mean absolute error; ML, machine learning; CV, cross-validation.

The processed dataset was used for model development and validation, as shown in Fig. 1. The patients included in the study were randomized into the training set and validation set at an 8:2 ratio. Based on previous literature [21, 22, 23], we selected both traditional linear models, such as the general linear model (GLM), and 10 commonly used ML algorithms to build the models, allowing for comparison between traditional and modern approaches. Specifically, these models included support vector machine with a linear kernel (SVM Linear), support vector machine with radial basis function kernel (SVM Radial), recursive partitioning and regression trees (RPART), gradient boosting machine (GBM), random forest (RF), generalized linear model with elastic net regularization (Glmnet), extreme gradient boosting with a linear booster (XGB Linear), kernel k-nearest neighbors (KKNN), convolutional neural network (CNN), and extreme gradient boosting (XGB). Considering the small size of the training and validation sets, we used 10-fold cross-validation (10-fold CV) for model evaluation and grid search for parameter optimization to ensure the robustness and accuracy of the model. In order to reduce the risk of overfitting, 10-fold CV was performed on the training set. In this process, the training set was first randomly divided into 10 subsets, with the model using 9 subsets as training data and the remaining subset as validation data for performance evaluation in each round. To further enhance the predictive performance of the model, grid search was used to optimize the model hyperparameters. The grid search method traverses all possible parameter combinations in the preset hyperparameter space, uses cross-validation to evaluate the performance of each set of hyperparameters, and finally selects the best hyperparameter combination. Specifically, hyperparameter optimization was performed for models such as SVM, RF, and XGB by adjusting parameters such as the C value, gamma value, number of trees, maximum depth, and learning rate to improve the model’s generalization and predictive accuracy. The best-performing model was selected after final evaluation, and its performance was validated using the validation set to ensure it had good generalization ability and clinical application potential. The parameter settings for the 10 ML algorithms are detailed in **Supplementary Table 1**.

### 2.8 Model Validation

To assess the predictive accuracy and performance of the model, the primary evaluation metrics used were mean absolute error (MAE) and R-squared (R^2^). R^2^ is the coefficient of determination (also known as goodness of fit) and represents the extent to which the regression model explains the variation in the dependent variable, or how well the model fits the observed data. The value of R^2^ ranges from 0 to 1. Generally, the closer R^2^ is to 1, the better the model fit, indicating a higher degree of explanation of the dependent variable by the independent variables. A higher R^2^ value implies that a larger percentage of the total variability is explained by the model. When the observed values are closer to the regression line, the fit is more accurate [24].

The MAE metric is used to evaluate the accuracy of a predictive model by calculating the average of the absolute differences between the actual values and the predicted values. Lower MAE and higher R^2^ indicate higher predictive accuracy, and thus better model performance.

Additionally, the ideal prediction percentage was used to evaluate the clinical utility of the model, defined as the percentage of predicted doses within ±20% of the actual dose. An increase in the ideal prediction percentage indicates greater clinical utility of the model.

A dose subgroup analysis was conducted to account for heterogeneity in clinical practice. The conventional warfarin dose in China is 2.5 mg/day. Based on clinical recommendations and existing studies [16, 17, 18], dose thresholds were defined as the 25th and 75th percentiles of the conventional dose, i.e., high-dose (≥3.125 mg/day), medium-dose (1.875–3.125 mg/day), and low-dose (≤1.875 mg/day). This categorization enables a more precise evaluation of the model’s efficacy across different dose levels, thereby optimizing clinical decision support.

### 2.9 Statistical Analysis

Continuous variables are presented as means ± standard deviations (SD) or median (interquartile range (IQR)), while categorical variables are presented as frequencies (n) and percentages (%). The normality of the continuous variables was assessed using the Shapiro-Wilk test. For comparisons of variance between groups, the F-test was applied to assess the homogeneity of variances. To compare continuous variables between two independent groups, the Student’s *t*-test was used when the data followed a normal distribution, while the Mann-Whitney U test was applied for non-normally distributed variables. For categorical variables, intergroup differences were assessed using the Chi-square test, with Fisher’s exact test applied when expected frequencies in any cell of the contingency table was less than 5.

Multiple ML techniques were utilized to model and analyze the data, with a variety of R packages employed to perform these analyses. These included the stats package (version 4.2.2; R Foundation for Statistical Computing, Vienna, Austria; https://www.R-project.org/) for basic statistical functions, e1071 (version 1.7-14; https://CRAN.R-project.org/package=e1071) for SVM implementations, RPART (version 4.1.23; https://CRAN.R-project.org/package=rpart) for recursive partitioning and decision tree algorithms, GBM (version 2.1.9; https://CRAN.R-project.org/package=gbm) for generalized boosting machines, RF (version 4.7-1.1; https://CRAN.R-project.org/package=randomForest) for ensemble learning, Glmnet (version 4.1-8; https://CRAN.R-project.org/package=glmnet) for penalized regression models, XGBoost (version 1.7.7.1; https://CRAN.R-project.org/package=xgboost) for gradient boosting machines, Class (version 7.3-22; https://CRAN.R-project.org/package=class) for classification algorithms, and Keras (version 2.15.0; https://CRAN.R-project.org/package=keras) for deep learning models. All statistical and machine learning analyses were performed using R (version 4.2.2; R Foundation for Statistical Computing, Vienna, Austria; https://www.R-project.org/). For all statistical tests, a significance threshold of *p*-value < 0.05 was considered to indicate statistical significance, and all tests were conducted with a two-sided approach.

## 3. Results

### 3.1 Baseline Characteristics of the Study Cohort

Following execution of the inclusion and exclusion criteria, a total of 413 patients were enrolled in the study, including 227 males (54.96%). The median age of participants was 53 (46–59) years, with an average height of 165.86 ± 8.37 cm, median weight of 65 (59–75) kg, average BSA of 1.75 ± 0.18 m^2^, and median BMI of 24.02 (22.04–27.03) kg/m^2^. The median stable daily warfarin dose was 3 (2.25–3.75) mg, and the median steady-state INR was 1.97 (1.78–2.20). A total of 332 patients were randomly selected as the training set (80%), while the remaining 81 patients were used as the validation set (20%). Baseline characteristics of the study participants are presented in Table 1. No significant differences in demographic baseline data or clinical and genetic information were found between the training and validation cohorts (all *p* > 0.05).

**Table 1.** **Characteristics of study participants**.

Characteristics, unit			Training set (N = 332)	Validation set (N = 81)	*p*-value
Demographic information					
	Age, years		52 (46–59)	54 (50–59)	0.220
	Sex, male, n (%)		186 (56.02)	41 (50.62)	0.381
	Height, cm		166.03 ± 8.34	165.16 ± 8.50	0.401
	Weight, kg		65.5 (59–75)	65 (59–75)	0.578
	BSA, m^2^		1.75 ± 0.18	1.74 ± 0.18	0.654
	BMI, kg/m^2^		24.02 (22.06–27.02)	24.22 (21.77–26.99)	0.844
	Smoke, n (%)		125 (37.65)	24 (29.63)	0.178
	Drink, n (%)		75 (22.59)	17 (20.99)	0.756
Comorbidities					
	Hypertension, n (%)		75 (22.59)	18 (22.22)	0.943
	Diabetes, n (%)		24 (7.23)	4 (4.94)	0.462
	Hyperlipidemia, n (%)		47 (14.16)	18 (22.22)	0.074
	Coronary artery disease, n (%)		21 (6.33)	7 (8.64)	0.457
	Cerebrovascular disease, n (%)		15 (4.52)	1 (1.23)	0.330*
	AF, n (%)		102 (30.72)	24 (29.63)	0.848
Combine medications					
	Digoxin, n (%)		57 (17.17)	11 (13.58)	0.435
	Amiodarone, n (%)		50 (15.06)	9 (11.11)	0.362
	Statin, n (%)		26 (7.83)	8 (9.88)	0.548
	ACEI, n (%)		12 (3.61)	1 (1.23)	0.478*
Preoperative test indicators					
	BNP, pg/mL		202.5 (75–479.75)	186 (65–317)	0.234
	ALT, U/L		17.5 (12–25)	17 (13–25)	0.596
	AST, U/L		19 (15–25)	20 (15–23)	0.906
	Creatinine, µmol/L		76.4 (66.65–90.2)	77.1 (65.1–87.8)	0.522
	Triglycerides, mmol/L		1.35 (1–1.88)	1.37 (1.01–1.91)	0.598
	TC, mmol/L		4.57 (3.92–5.29)	4.77 (4.12–5.54)	0.082
	HDL, mmol/L		1.15 (0.92–1.34)	1.15 (0.94–1.32)	0.982
	LDL, mmol/L		2.76 (2.16–3.43)	2.89 (2.46–3.53)	0.129
	Prothrombin Time, s		11.8 (11.2–12.75)	11.6 (11.1–12.9)	0.784
	INR		1.04 (1–1.14)	1.04 (1–1.17)	0.783
	Fibrinogen, g/L		2.8 (2.3–3.2)	2.7 (2.4–3.3)	0.900
Preoperative echocardiogram					
	EF, %		60 (55–64.25)	60 (54–65)	0.637
	LVEDD, mm		51 (46–60.25)	50 (45–56)	0.242
	LVESD, mm		35 (30–42)	35 (30–39)	0.264
	LAD, mm		58 (47–68)	57 (50–64)	0.679
NYHA classification					0.284
	Level I, n (%)		10 (3.01)	3 (3.70)	
	Level II, n (%)		123 (37.05)	21 (25.93)	
	Level III, n (%)		161 (48.49)	50 (61.73)	
	Level IV, n (%)		38 (11.45)	7 (8.64)	
Surgery type					
	Mitral valve replacement, n (%)		114 (34.34)	33 (40.74)	0.281
	Aortic valve replacement, n (%)		151 (45.48)	34 (41.98)	0.569
	Double valve replacement, n (%)		67 (20.18)	14 (17.28)	0.556
	Combined Cox-Maze procedure, n (%)		51 (15.36)	8 (9.88)	0.206
	Warfarin maintenance dose, mg/day		3 (2.25–3.38)	3 (2.25–3.75)	0.641
	Steady-state INR		1.96 (1.78–2.2)	2.03 (1.8–2.2)	0.261
Genetic profiles					
	*CYP2C9* (rs1057910), n (%)				0.979
		*1/*1	303 (91.27)	74 (91.36)	
		*1/*3	29 (8.73)	7 (8.64)	
	*VKORC1* (rs9923231), n (%)				0.166*
		*AA*	278 (83.73)	66 (81.48)	
		*AG*	53 (15.96)	13 (16.05)	
		*GG*	1 (0.31)	2 (2.47)	
	*CYP4F2* (rs2108622), n (%)				0.267*
		*CC*	182 (54.82)	41 (50.62)	
		*CT*	133 (40.06)	32 (39.51)	
		*TT*	17 (5.12)	8 (9.87)	
	*APOE *(rs7412), n (%)				1.000*
		*CC*	273 (82.23)	68 (83.95)	
		*CT*	56 (16.87)	13 (16.05)	
		*TT*	3 (0.90)	0	
	*CYP1A2* (rs2069514), n (%)				0.664*
		*AA*	278 (83.73)	65 (80.25)	
		*AG*	48 (14.46)	15 (18.52)	
		*GG*	6 (1.81)	1 (1.23)	
	*CYP3A4 *(rs28371759), n (%)				0.474*
		*AA*	321 (96.69)	80 (98.77)	
		*AG*	11 (3.31)	1 (1.23)	

Data are presented as the mean ± SD, median (interquartile range, IQR) and n (%). * denotes Fisher’s exact test. BSA, body surface area; BMI, body mass index; AF, atrial fibrillation; ACEI, angiotensin converting enzyme inhibitors; BNP, B-type natriuretic peptide; ALT, alanine transaminase; AST, aspartate aminotransferase; TC, total cholesterol; HDL, high density lipoprotein; LDL, low density lipoprotein; INR, international normalized ratio; EF, ejection fraction; LVEDD, left ventricular end-diastolic dimension; LVESD, left ventricular end-systolic dimension; LAD, left atrial diameter; NYHA, New York Heart Association.

### 3.2 Key Features of the Model

ANCOVA was used to select variables from the potential independent factors. Based on these criteria, 13 primary input variables were chosen from the initial factors for subsequent construction of the model (Table 2), namely sex, age, BSA, height, weight, smoke, digoxin, AF, TC, LDL, Cox-Maze,* VKORC1*, and *CYP2C9*. Among the variables included in the model, AF had the largest partial η^2^ value (0.016), sex and TC had the smallest partial η^2^ values (0.002), while the remaining variables had partial η^2^ values ranging from 0.003 to 0.012.

**Table 2.** **Input variables**.

Included variables, unit	Partial η²
Sex	0.002
Age, years	0.003
BSA, m^2^	0.005
Weight, kg	0.004
Height, cm	0.004
Smoke	0.011
Digoxin	0.011
AF	0.016
TC, mmol/L	0.002
LDL, mmol/L	0.003
Cox-Maze	0.012
*VKORC1*	0.007
*CYP2C9*	0.009

Partial η^2^ indicates the explanatory power of the independent variables on the dependent variable. Considering the conciseness of the model, the threshold value of η^2^ was set as 0.002. If it was ≥0.002, it was included as an input variable. BSA, body surface area; AF, atrial fibrillation; TC, total cholesterol; LDL, low density lipoprotein.

### 3.3 Comprehensive Comparison of Prediction Algorithms

**Fig. 2.**
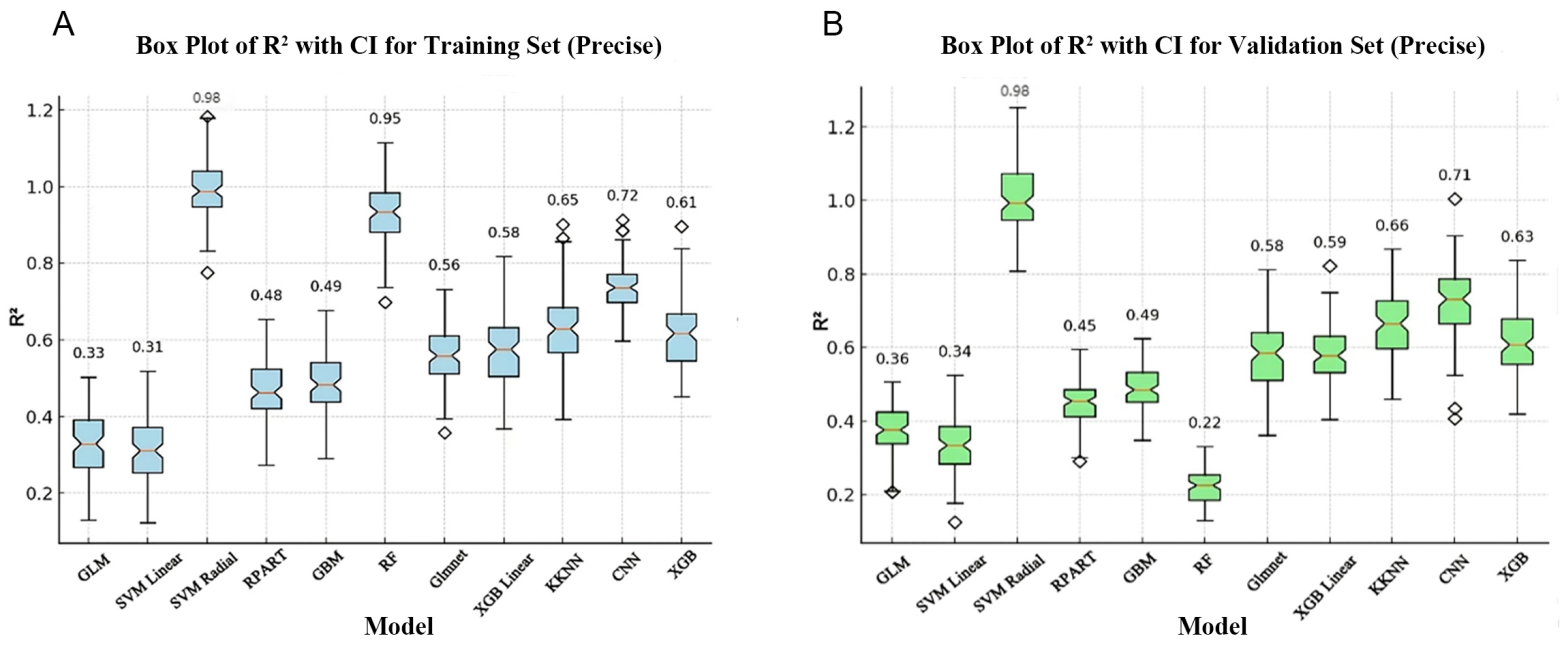
**Comparison of R^2^ for the training and validation sets for different algorithms**. (A) Box plot of R^2^ values for the training set. (B) Box plot of R^2^ values for the validation set. The horizontal axis represents the different models and the vertical axis represents the R^2^ values. The box plot shows the median, interquartile range and 95% confidence interval (CI) of the R^2^ values. GLM, general linear model; SVM Linear, support vector machine with linear kernel; SVM Radial, support vector machine with radial basis function kernel; RPART, recursive partitioning and regression trees; GBM, gradient boosting machine; RF, random forest; Glmnet, generalized linear model with elastic net regularization; XGB Linear, extreme gradient boosting with linear booster; KKNN, kernel k-nearest neighbors; CNN, convolutional neural network; XGB, extreme gradient boosting.

To assess the predictive accuracy and performance of the models, we first evaluated the R^2^ of 10 ML models and of the GLM model. In the training set, the SVM Radial model and the RF model have the highest R^2^ of 0.98 and 0.95, respectively, while the SVM Linear model and the GLM model have the lowest R^2^ of 0.31 and 0.33, respectively. The remaining models have R^2^ ranging between 0.48 and 0.72 (Fig. 2A). In the validation set, the SVM Radial model had the highest R^2^ of 0.98, demonstrating high stability and generalization ability, and indicating it has significant advantages in terms of handling complex data patterns and lower susceptibility to overfitting (Fig. 2B). However, RF had the lowest R^2^ (0.22) in the validation set (Fig. 2B), performing noticeably worse than in the training set. This result suggests potential overfitting, where the model learns too many details and noise from the training data that do not apply to new, unseen data. The R^2^ for the remaining models with the validation set ranged from 0.34 to 0.71 (Fig. 2B).

Next, we calculated the MAE of all models. The MAE of SVM Radial was the lowest at 0.14 mg/day (95% CI: 0.11–0.17), indicating it was a stable model. In contrast, the MAE in the validation set was highest in RF at 0.93 mg/day (95% CI: 0.88–0.98) (Table 3). This indicates RF was an unstable model, probably due to the small sample size and overfitting.

**Table 3.** **MAE of different algorithms in the validation set**.

Model	MAE (95% CI), mg/day
GLM	0.74 (0.68–0.80)
SVM Linear	0.77 (0.71–0.83)
SVM Radial	0.14 (0.11–0.17)
RPART	0.24 (0.19–0.28)
GBM	0.47 (0.42–0.52)
RF	0.93 (0.88–0.98)
Glmnet	0.75 (0.71–0.79)
XGB Linear	0.76 (0.71–0.81)
KKNN	0.21 (0.17–0.25)
CNN	0.39 (0.33–0.45)
XGB	0.35 (0.30–0.40)

MAE, mean absolute error; 95% CI, 95% confidence interval; GLM, general linear model; SVM Linear, support vector machine with linear kernel; SVM Radial, support vector machine with radial basis function kernel; RPART, recursive partitioning and regression trees; GBM, gradient boosting machine; RF, random forest; Glmnet, generalized linear model with elastic net regularization; XGB Linear, extreme gradient boosting with linear booster; KKNN, kernel k-nearest neighbors; CNN, convolutional neural network; XGB, extreme gradient boosting.

**Fig. 3.**
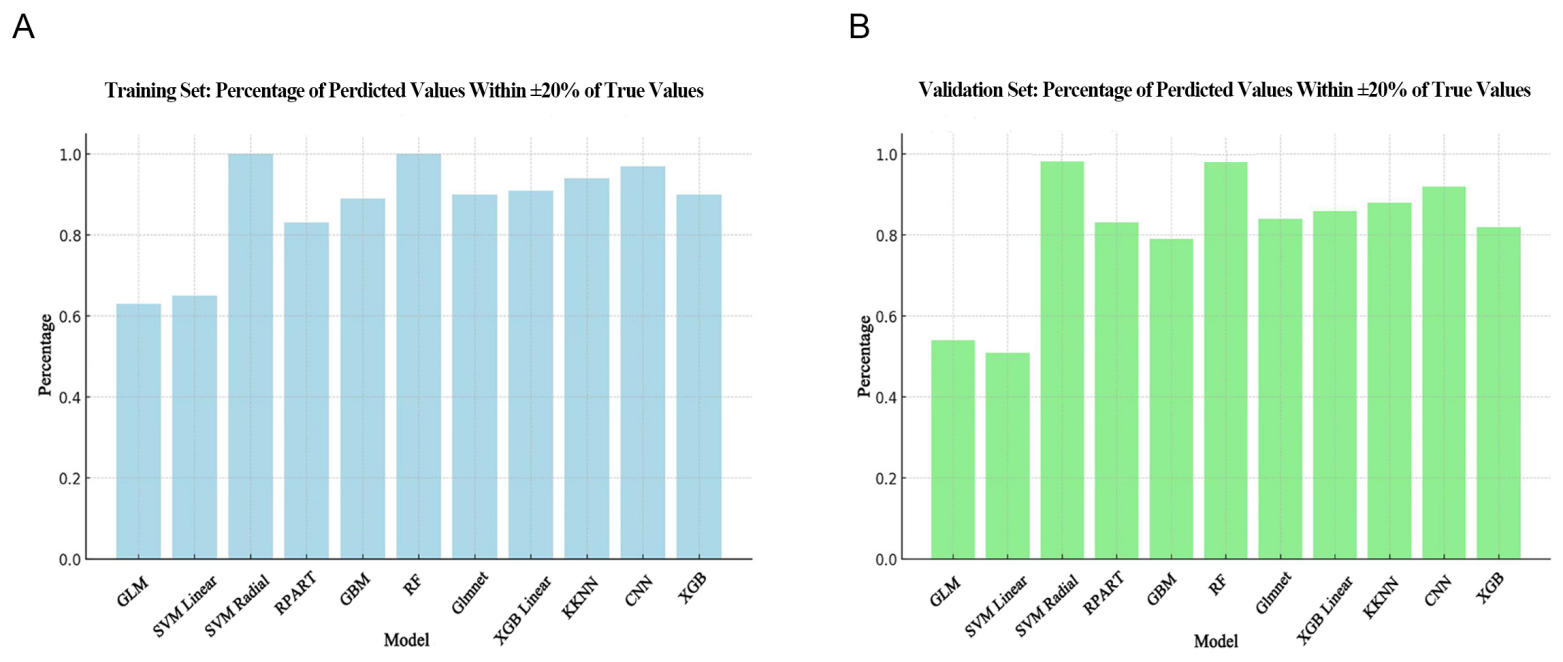
**Comparison of ideal prediction percentage for different algorithms**. (A) Ideal prediction percentage in the training set. (B) Ideal prediction percentage in the validation set. The horizontal axis shows the different models, while the vertical axis shows the proportion of samples where the predicted value is within ±20% of the true value. GLM, general linear model; SVM Linear, support vector machine with linear kernel; SVM Radial, support vector machine with radial basis function kernel; RPART, recursive partitioning and regression trees; GBM, gradient boosting machine; RF, random forest; Glmnet, generalized linear model with elastic net regularization; XGB Linear, extreme gradient boosting with linear booster; KKNN, kernel k-nearest neighbors; CNN, convolutional neural network; XGB, extreme gradient boosting.

Calculation of the ideal prediction percentage revealed the SVM Radial and RF models performed best. Moreover, the results obtained with the validation set (Fig. 3B) for these two models were as good as with the training set (Fig. 3A). The lowest ideal prediction percentage observed in the training set was GLM, while in the validation set it was SVM Linear.

In summary, the SVM Radial model demonstrated the highest R^2^, the best ideal prediction percentage, and the smallest MAE. We therefore selected SVM Radial as the optimal model for predicting stable warfarin dose in patients following surgery for mechanical heart valve replacement.

### 3.4 Clinical Significance

**Fig. 4.**
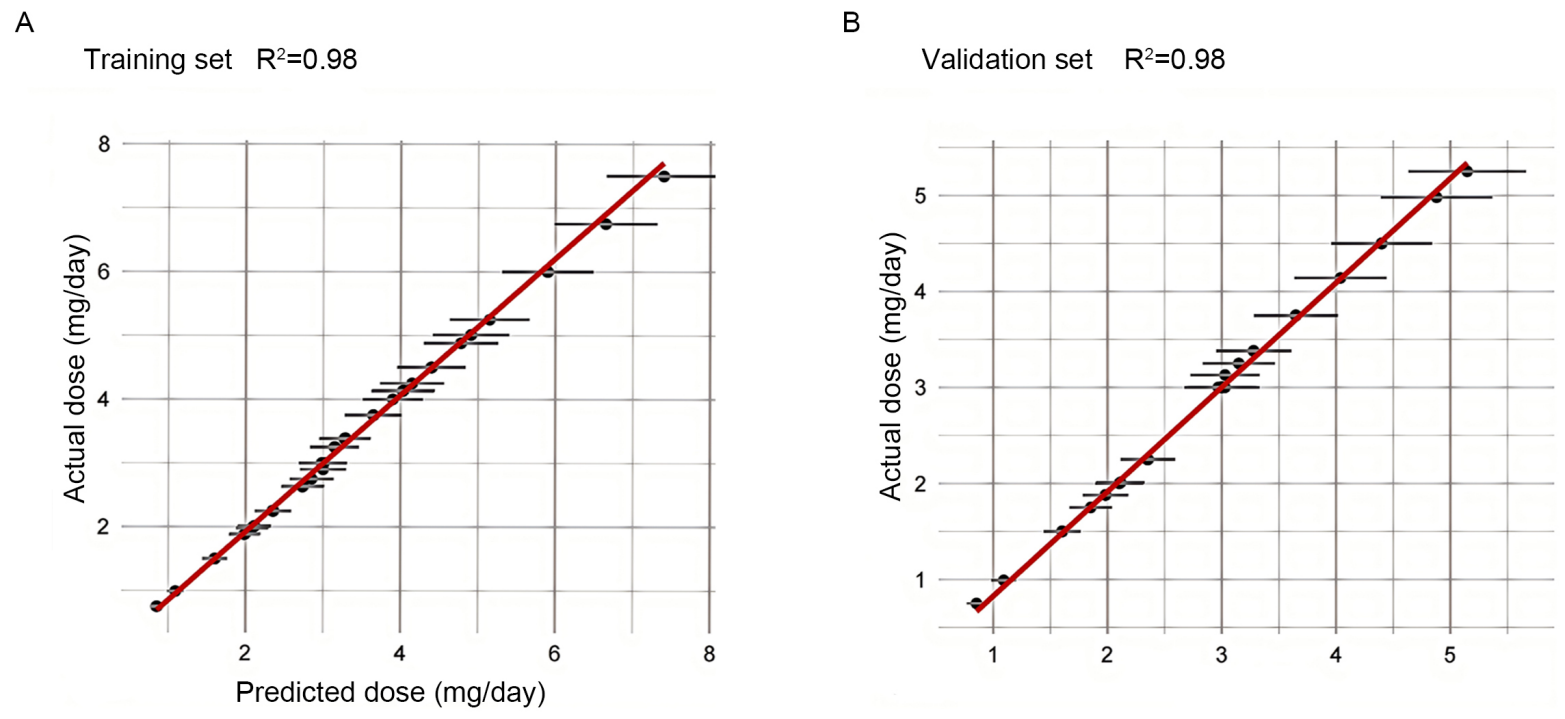
**Plot of predicted versus actual dose fit assessment for the training and validation sets**. (A) Plot of predicted versus actual dose fit assessment for the training set. (B) Plot of predicted versus actual dose fit assessment for the validation set. The horizontal axis shows the predicted dose, and the vertical axis shows the actual dose. The solid red line indicates the ideal fit (y = x), and the black dots represent the median of the actual values within each prediction interval, while the error bars reflect the variability of the data within that interval.

To further evaluate the overall predictive performance of the SVM Radial model, we plotted the predicted dose versus actual dose fit assessment in both the training and validation datasets (Fig. 4). In the training and validation sets, R^2^ was 0.98, indicating a strong correlation between the predicted values and the actual observations, and demonstrating the excellent predictive capabilities of the SVM Radial model.

In the dose subgroup analysis, the SVM Radial model demonstrated high accuracy for predicting warfarin doses. For the total sample, this model successfully predicted the ideal dose in 98.06% of cases. A detailed analysis of different dose levels revealed a prediction success rate of 95.00% for the 40 subjects requiring a low daily dose (≤1.875 mg/day), 98.78% for the 245 subjects in the medium-dose group (1.875–3.125 mg/day), and 97.66% for 128 subjects in the high-dose group (≥3.125 mg/day) (Table 4).

**Table 4.** **The predictive ability of SVM Radial in different dose subgroups**.

Actual dose		N	Ideal (%)	Underestimate (%)	Overestimate (%)
Total cohort		413	405 (98.06)	7 (1.70)	1 (0.24)
	≤1.875 mg/day	40 (9.69)	38 (95.00)	1 (2.50)	1 (2.50)
	1.875–3.125 mg/day	245 (59.32)	242 (98.78)	3 (1.22)	0
	≥3.125 mg/day	128 (30.99)	125 (97.66)	3 (2.34)	0
Validation cohort		81	76 (93.83)	4 (4.94)	1 (1.23)
	≤1.875 mg/day	7 (8.65)	6 (85.71)	0	1 (14.29)
	1.875–3.125 mg/day	49 (60.49)	47 (95.92)	2 (4.08)	0
	≥3.125 mg/day	25 (30.86)	23 (92.00)	2 (8.00)	0

Data are presented as n (%). The total cohort includes the training cohort and validation cohort. Ideal percentage of patients whose predicted absolute error between the predicted and actual doses was within 20% of the actual dose. Underestimate percentage of patients whose predicted dose was less than the actual dose, and the predicted absolute error between the predicted and actual doses was <20% of the actual dose. Overestimate percentage of patients whose predicted dose was more than the actual dose, and the predicted absolute error between the predicted and actual doses was >20% of the actual dose. SVM Radial, support vector machine with radial basis function kernel.

The SVM Radial model accurately predicted the dose for 93.83% of the 81 subjects in the validation cohort. In the different dose groups, the prediction accuracy was 85.71% for 7 subjects in the low-dose group (≤1.875 mg/day), 95.92% for 49 subjects in the medium-dose group (1.875–3.125 mg/day), and 92.00% for 25 subjects in the high-dose group (≥3.125 mg/day). These results demonstrate the superior performance of the SVM Radial model across various dose levels and highlight its potential value in clinical applications. In particular, this model has excellent predictive ability within the medium-dose range, providing guidance for precise dose adjustment in clinical practice.

## 4. Discussion 

Our study had the following important features. First, this study was based on case data from the Beijing Anzhen Hospital, recognized as a highly authoritative cardiovascular center in China. We included a wide range of clinical and genetic factors in our model construction to broaden its array of features. The genetic factors consisted of six SNP loci whose inclusion further enhance the model’s predictive performance and have not been included in most previous studies. Second, due to the relatively small sample size of this study, we also evaluated the performance of 10 artificial intelligence ML methods and selected the best-performing model to ensure predictive accuracy. Third, the median warfarin maintenance dose in our study was 3 mg/day, which is lower than the 4 mg/day maintenance dose used in the IWPC model [8]. The target INR range was set at 1.5–2.5, which is significantly lower than the internationally used range of 2.0–3.0. The lower the intensity of anticoagulation, the lower the warfarin dose. Research has shown that in Chinese patients undergoing heart valve replacement surgery, low-intensity anticoagulation therapy (INR 1.5–2.5) does not increase the incidence of embolic or thrombotic complications, but significantly reduces the risk of bleeding complications and mortality [25]. Therefore, our algorithm is particularly suitable for individualized warfarin anticoagulation treatment of Chinese patients following heart valve surgery. By implementing this individualized dosing model, patient safety can be enhanced by minimizing the risk of adverse events such as bleeding or thrombosis. Additionally, the need for frequent dose adjustments is reduced, thereby making the overall management of warfarin therapy more efficient.

Robust evidence already exists in the literature regarding factors that influence the stable maintenance dose of warfarin investigated in this study. Height, weight, and BSA were positively correlated with warfarin dosage. Age, height, and weight have been reported to account for 76.8% of the variations in warfarin dosage [26], with most researchers agreeing that older adults require lower doses [27]. This might be due to reduced hepatic blood flow with increasing age, resulting in decreased warfarin transport to the liver and possibly diminished vitamin K absorption in elderly patients [28]. AF is positively correlated with warfarin dose, and the relatively high intensity of anticoagulation in targets with combined AF leads to an increase in the required dose [29]. The Cox-Maze procedure is a viable technique for treating heart valve disease combined with AF [30]. To minimize bleeding and thromboembolic complications, radiofrequency ablation is usually performed concurrently with valve replacement, thus requiring optimal perioperative anticoagulation with warfarin [31]. With regard to concomitant medications, amiodarone is one of the most frequently included features in previous warfarin dosing prediction models. However, it was not included as a key variable in the present study due to its relatively small weight. Our study did however incorporate digoxin, which competitively binds to P-glycoprotein to reduce the transport and elimination of warfarin, thereby enhancing the drug concentration and anticoagulant effect [32]. Furthermore, our study suggests that inclusion of TC and LDL levels may lead to the development of models with higher prediction accuracy. A possible underlying mechanism is that high cholesterol and elevated LDL levels are associated with fatty liver, which can impact the fluidity of liver cell membranes and affect the cytochrome P450 enzyme system, particularly CYP2C9, thereby influencing warfarin metabolism [33]. Additionally, high cholesterol may alter plasma protein levels [34], affecting the binding of warfarin to albumin and its free fraction, and thus modifying its anticoagulant effect [35]. Elevated cholesterol may also increase inflammatory markers such as C-reactive protein (CRP), potentially altering the patient’s coagulation status and reducing the efficacy of warfarin [36].

*VKORC1* and *CYP2C9* have been reported to account for approximately 35%–50% of the variability in warfarin dose requirements [37]. These genes are widely recognized for their significant influence on the warfarin dosage. *VKORC1* is a rate-limiting enzyme in the vitamin K cycle and the activation of clotting factors. It affects warfarin pharmacodynamics through its interaction with various warfarin targets [38]. Significant differences have been reported in the frequency of *VKORC1* genotypes between different races. Most of the Chinese Han population carry the *VKORC1* mutant genotype (*AA/AG*), which requires a lower warfarin dose compared to wild-type (*GG*) patients [6]. *CYP2C9* is a key drug-metabolizing enzyme found primarily in the liver. It catalyzes the conversion of S-warfarin to its metabolites, altering the stable therapeutic dose by influencing warfarin metabolism. The *3 variant of *CYP2C9* is the most prevalent variant in the Chinese population [39], and frequencies of the CYP2C9 *1/*1, *1/*3 and *3/*3 genotypes in the Chinese Han population are 91.0%, 8.44% and 0.55%, respectively [40]. Their frequencies in the current study (91.28%, 8.72% and 0, respectively) concur with the previous results. Compared to CYP2C9*1 homozygous patients, those harboring the *3 mutation require only a low dose of warfarin to achieve the same anticoagulant effect [41]. Authoritative dosing algorithms, such as IWPC [8] and Gage [9], have demonstrated that genetic data can significantly enhance the predictive performance of these models, explaining why our algorithm also has high predictive accuracy.

The MAE of our SVM Radial model was 0.14 mg/day, or close to 0.98 mg/week. This is considerably lower than the IWPC genetic model of 8.5 mg/week [8]. Moreover, the SVM Radial model had a higher R^2^ value (0.98 vs. 0.43). In the dose subgroup analyses, the SVM Radial model had a higher ideal prediction percentage compared to the IWPC model in the low-dose (85.71% vs. 33.00%, respectively), medium-dose (95.92% vs. 54.60%), and high-dose (92.00% vs. 36.80%) subgroups, thus demonstrating better predictive performance. The predictive accuracy of the ML algorithm was significantly improved compared to MLR.

Several ML algorithms have been used for warfarin dose prediction. Tao *et al*. [42] built an adaptive neuro-fuzzy inference system (ANFIS). Our SVM Radial model outperformed the ANFIS model in terms of ideal prediction percentage (93.83% vs. 63.7%, respectively) and MAE (0.140 vs. 0.603). Li *et al*. [16] developed a back-propagation neural network (BPNN) model to predict the maintenance dose of warfarin after heart valve surgery. The BPNN model had a higher MAE value than the SVM Radial model (0.614 vs. 0.140, respectively), and worse performance on the ideal prediction percentage (47.86% vs. 93.83%). The strength of both the Tao *et al*. [42] and Li *et al*. [16] studies was their large sample size of >10,000 patients. Ma *et al*. [43] later applied equal stratified sampling to build a feed-forward neural network (FFNN) model for the prediction of warfarin dose. The MAE of this model was higher than that of SVM Radial (0.345 vs. 0.140, respectively), but the ideal prediction percentage reached 95.6% in the medium-dose group, which was similar to that of SVM Radial (95.92%). The FFNN model also performed well in terms of the ideal prediction percentage in the low-dose (70.2%) and high-dose (75.1%) subgroups, demonstrating the effectiveness of hierarchical training in improving the prediction accuracy of the model. However, none of the above ML algorithms incorporate genetic information such as SNP data, which may affect the accuracy of model prediction. In the studies published to date, the prediction accuracies of the models were all significantly higher in the medium-dose group than in the low- and high-dose groups, which was caused by the larger proportion of the patient population in the medium-dose group [15, 16, 17, 18]. However, the hybrid model with genetic algorithm and Back Propagation neural network (BP-GA) established by Li *et al*. [17] showed very low prediction accuracies in both the low-dose group (the warfarin dose was overestimated in >98% of patients) and the high-dose group (the warfarin dose was underestimated in >98% of patients). Such miscalculations may lead to overdosing or underdosing of warfarin, thus increasing the risk of serious bleeding or thrombosis [2]. An increasing number of studies have therefore emphasized the need to improve model performance in extreme dose groups (low- and high-dose subgroups) [16, 17, 18]. In the present study, the SVM Radial model demonstrated strong predictive accuracy in both the low-dose (85.71%) and high-dose (92.00%) groups. These results highlight the ability of our screened dosage model to recommend appropriate warfarin dosages that are beneficial to the vast majority of subjects, with good predictive utility.

There were some limitations to this study. Firstly, the construction of our model was based on a specific population of post-surgical patients who underwent mechanical heart valve replacement in a single center. The results were highly specific and sensitive, thus limiting their generalizability and universality to different patient groups, especially in high-risk patient populations. Future studies should include a large sample size to ensure diversity and representativeness of the training data, and to improve the customization of high-risk patient groups. Second, this was a retrospective study with a relatively small sample size and lack of external validation. Future studies should aim to increase the sample size, recruit more subjects, have a prospective study design, and include multicenter validation to test the predictive performance of any new models. Meanwhile, the models should be continuously optimized and updated by including new clinical data and multicenter data. They should be dynamically adjusted to maintain the accuracy of prediction and to ensure the model is capable of adapting to changing clinical needs. Due to the unpopularity and expense of genetic testing in developing countries and the complexity of ML algorithms, the practical application of these models is still limited by their high implementation cost, complex operational requirements, high patient compliance requirement, and high demand for medical resources. By integrating SHapley Additive exPlanations (SHAP) or Local Interpretable Model-agnostic Explanations (LIME) tools [44, 45], model interpretability can be enhanced, making decision-making processes more transparent and fostering greater trust and adoption. Alternatively, developing hybrid pharmacokinetic (PK)/pharmacodynamic (PD)-ML models [46] can enable more precise, individualized healthcare, improving medical efficiency and further boosting the clinical utility of these models in the future.

## 5. Conclusions

The SVM Radial model based on clinical and genetic factors demonstrated high predictive accuracy, especially in low- and high-dose groups. This model shows great promise in predicting the appropriate maintenance dose of warfarin after surgery for mechanical heart valve replacement. However, further validation through large, multicenter prospective studies is necessary to improve the generalizability and practical application of the SVM Radial model in different patient populations.

## Availability of Data and Materials

The datasets used and analyzed during the current study are available from the corresponding author on reasonable request.

## Supplementary Material

Supplementary material associated with this article can be found, in the online version, at https://doi.org/10.31083/RCM33425.
