# Supplementary material for: A Prediction Model of Stable Warfarin Doses in Patients After Mechanical Heart Valve Replacement Based on a Machine Learning Algorithm
Source: Rev Cardiovasc Med. 2025 Jun 26;26(6):33425. doi: 10.31083/RCM33425 (PMC12230826; doi:10.31083/RCM33425)
Supplement: Supplementary file 1 [file 2153-8174-26-6-33425-s1.docx]

**Supplementary Table 1 Parameter settings of 10 ML Algorithms**

| ML algorithms | **Parameter settings** |
| --- | --- |
| SVM Linear | kernel = "linear", cost = 1, type = "C-classification" |
| SVM Radial | kernel = "radial", cost = 20, gamma = 0.3,  type = "eps-regression" |
| RPART | method = "poisson", cp = 0.01, minsplit = 20, maxdepth = 10 |
| GBM | n.trees = 5000, shrinkage = 0.01, interaction.depth = 3, n.minobsinnode = 10 |
| RF | n_estimators = 1000, max_depth = None, min_samples_split = 2, random_state = 42, proximity=TRUE,mtry = 8 |
| Glmnet | alpha = 1, lambda = 0.01 |
| XGB Linear | eta = 0.3, lambda = 1, alpha = 0 |
| KKNN | k = 8, distance = 2, kernel = "rectangular" |
| CNN | layers = [32, 64, 128], activation = ReLU, optimizer = adam, epochs = 50, batch_size = 32 |
| XGB | booster = "gbtree",eval_metric = "error",gamma = 0.3,eta = 0.3, max_depth = 6, n_estimators = 100, subsample = 0.8, colsample_bytree = 0.8,max_depth = 5,subsample = 1,colsample_bytree = 2 |

ML, machine learning; SVM Linear, support vector machine with linear kernel; SVM Radial, support vector machine with radial basis function kernel; RPART, recursive partitioning and regression trees; GBM, gradient boosting machine; RF, random forest; Glmnet, generalized linear model with elastic net regularization; XGB, Linear extreme gradient boosting with linear booster; KKNN, kernel k-nearest neighbors; CNN, convolutional neural network; XGB, extreme gradient boosting
